# Serial Non-Invasive Monitoring of Renal Disease Following Immune-Mediated Injury Using Near-Infrared Optical Imaging

**DOI:** 10.1371/journal.pone.0043941

**Published:** 2012-09-26

**Authors:** Yong Du, Shion An, Li Liu, Li Li, Xin J. Zhou, Ralph P. Mason, Chandra Mohan

**Affiliations:** 1 Internal Medicine/Rheumatology, University of Texas, Southwestern Medical Center, Dallas, Texas, United States of America; 2 Radiology, University of Texas, Southwestern Medical Center, Dallas, Texas, United States of America; National Cancer Institute, United States of America

## Abstract

**Background:**

Non-invasive monitoring of disease progression in kidney disease is still a major challenge in clinical practice. *In vivo* near-infrared (NIR) imaging provides a new tool for studying disease mechanisms and non-invasive monitoring of disease development, even in deep organs. The LI-COR IRDye® 800CW RGD optical probe (RGD probe) is a NIR fluorophore, that can target integrin alpha v beta 3 (α_v_β_3_) in tissues.

**Objective:**

This study aims to monitor renal disease progression in an anti-glomerular basement membrane (GBM) nephritis mouse model.

**Methods:**

Anti-GBM nephritis was induced in 129x1/svJ mice by anti-GBM serum challenge. The expression of integrin α_v_β_3_ in the diseased kidney was examined by immunohistochemistry and quantitative polymerase chain reaction. The RGD probe and control fluorophores, the 800CW dye, and the BSA-conjugated 800CW dye, were administered into anti-GBM nephritic mice. LI-COR Pearl® Impulse imaging system was used for *in vivo* imaging; while *ex vivo* organ imaging was acquired using the Maestro^TM^ imaging system.

**Results:**

Kidney tissue from anti-GBM nephritic mice showed higher levels of integrin α_v_β_3_ expression at both the protein and the mRNA level compared to normal mice. The RGD probe allowed *in vivo* renal imaging and the fluorescent signal could be specifically captured in the diseased kidneys up to 14 days, reflecting longitudinal changes in renal function.

**Conclusion:**

The infrared RGD molecular probe that tracks integrin expression can be successfully used to monitor renal disease progression following immune-mediated nephritis.

## Introduction

Acute kidney injury (AKI) is a common problem, affecting more than 2 million people worldwide each year. Despite significant advances in both technology and clinical care, the mortality and morbidity rate associated with AKI has remained relatively unchanged at around 50% over the past four decades, alluding to shortfalls in early diagnosis, disease monitoring as well as therapy [1]. Although there is a wealth of evidence indicating that new molecular biomarkers, such as neutrophil gelatinase-associated lipocalin [2], [3] and IL-18 [2], [4], can be used to aid in AKI diagnosis and/or non-invasively monitor disease progression, these studies are still preliminary and need further studies to validate the sensitivity and specificity of these molecules in larger cohorts [2], [5]. Therefore, the development of a non-invasive tool to monitor renal disease as well as to guide treatment decision is urgently warranted.

*In vivo* imaging has recently emerged in medical research as an effective approach to non-invasively monitor molecular mechanisms and disease progression, providing both qualitative and quantitative data. Magnetic resonance imaging (MRI) has successfully been used to quantify renal inflammation in MRL/lpr mice [6], to detect renal involvement in a murine lupus model or lupus [7], [8], to identify and differentiate various types of nephropathies [9] as well as to evaluate glomerular filtration rate (GFR) [10]. *In vivo* fluorescent imaging has rarely been used for this imaging purpose, since the kidney is a deep organ and traditional fluorophores have limited tissue light penetration [11], [12]. New near infrared (NIR) fluorophores offer enhanced tissue penetration. These NIR fluorochromes have high molar extinction coefficients, good quantum yields, and low non-specific tissue binding, which makes deep-organ *in vivo* imaging possible [11]–[15]. Recently, Nakamura *et al*. have applied *in vivo* optical imaging in three different renal disease models using Cy7-labeled recombinant-gelatin (R-Gel). Their data indicated that this probe accumulated at the site of inflammation within the diseased kidney, with a pattern similar to that obtained by fluorescent imaging following the administration of anti-Mac1 antibody [16]. In a rat model of polycystic kidney disease, GFR was successfully tracked by optical imaging with the fluorescent renal marker fluorescein-isothiocyanate-labeled-sinistrin [10].

Integrin, a heterodimeric transmembrane receptor glycoprotein with α and β subunits, plays a critical role in mediating adhesion and interaction between cells and the extracellular matrix. Early studies demonstrated that alpha v beta 3 (αvβ3) is highly expressed in various forms of glomerulonephritis, including IgA nephropathy, lupus nephritis, membranoproliferative glomerulonephritis, as well as diabetic nephropathy [17]–[21]. The distribution of integrin αvβ3 is observed mainly around the expanded mesangial regions in close proximity to the immune complex deposits as well as in glomerular capillary loops and cellular crescents. More importantly, expression has been shown to increase significantly as a degree of chronic histological damage and disease progression [18], [20], [21].

RGD is a small peptide with an Arg-Gly-Asp sequence, which exhibits high-affinity binding to the αvβ3 integrin [22], [23]. IRDye 800CW RGD (LI-COR Biosciences), a NIR dye conjugated to RGD, has recently been developed and used for *in vivo* optical cancer imaging. After administration, the dye is distributed and excreted by the kidneys, without any obvious adverse effects on renal function [24]. Considering the advantages of NIR fluorophores and the high affinity of the RGD probe for kidneys expressing integrin αvβ3, we selected the LI-COR 800CW RGD molecular probe (RGD probe) as our targeting contrast agent to monitor renal disease progression in a mouse model with anti-glomerular basement membrane antibody (anti-GBM)- induced experimental nephritis.

Anti-GBM nephritis is a typical AKI model, characterized by rapid progressive renal dysfunction and aggressive crescent formation. This disease is regarded as the prototype of immune complex-induced nephritis because of the presence of the anti-GBM autoantibody and the successful identification of auto-antigens on the renal GBM [25], [26]. However, increasing evidence indicates that multiple factors such as chemokines, cytokines, T cells, as well as genetic factors also contribute to the pathogenesis of the disease [27]. Similar to other types of glomerulonephritis, elevated expression of αvβ3 has also been demonstrated in human crescentic glomerulonephritis [19].

In the present study, we aim to apply non-invasive *in vivo* optical renal imaging using the LI-COR RGD probe to track disease in a mouse model of anti-GBM nephritis. Our strategy offers promise as a non-invasive method to monitor renal disease. Most importantly, this strategy has the potential for targeted monitoring and treatment of various renal diseases in the near future.

## Materials and Methods

### Anti-GBM nephritis model

Among 20 inbred strains, 129x1/svJ mice are one of the most susceptible strains to anti-GBM disease [28]. The methodology to create the experimental anti-GBM nephritis model has been described elsewhere [28]. Briefly, 5 days after immunization with rabbit IgG in complete Freund’s adjuvant (Sigma, St. Louis, MO), anti-GBM serum was administered intravenously to 30 129x1/svJ mice (6–8 wks old). The imaging was initiated on Day 12 (D12) after immunization on Day 0 (D0), when disease begins in this model, as evidenced by higher levels of serum creatinine (Scr) and urine protein, as well as increased pathological injury. The pathological changes have previously been evaluated by a renal pathologist in a blind fashion, and the detailed scoring system has been described elsewhere [28]. All studies were reviewed and approved by the Institutional Animal Care and Use Committee at UT Southwestern Medical Center, Dallas, TX.

### The expression of integrin αvβ3 in nephritis

The expression of α_v_β_3_ was evaluated by IHC. Briefly, after initial dewaxing and rehydration of 4-µm formalin-fixed, paraffin-embedded kidney sections from both anti-GBM nephritis and normal 129x1/svJ mice, antigen retrieval was performed in a citrate buffer (pH 6.0). Then, rabbit anti-mouse α_v_β_3_ was incubated overnight at 4°C (1:500; Abbiotec, San Diego, CA). EXPOSE rabbit and mouse specific IHC kit (Abcam Inc, Cambridge, MA) was used for the detection of integrin α_v_β_3_ expression. The sections were counterstained with hematoxylin to visualize cell nuclei.

Quantitative polymerase chain reaction (qPCR) was performed to detect integrin αvβ3 mRNA expression in the kidney tissue. The primer sequence for integrin αv was 5′-GGTTACTCTTGGCTTAGGTGG-3′ upstream and 5′- CTTCCCTGCTAGTTTCCTCATC-3′ downstream. For integrin β3, the primer sequence was 5′-GCTCATCGTTTCTATCCCACC-3′ upstream and 5′-TTCATCGGGTTTCCAAGGTC-3′ downstream. After isolating total RNA from kidney tissue using TRIzol, cDNA was generated using the TaqMan reverse transcription reagent (Applied Biosystems, Foster City, CA). mRNA levels were determined by real-time PCR using the Fast SYBR® Green Master Mix (Applied Biosystems) on a Bio-Rad CFX96™ real-time system (Bio-Rad, Hercules, CA). Expression of all genes was normalized to cyclophilin A-2 expression using the standard ΔC_T_ method.

### Conjugation of bovine serum albumin (BSA) with 800CW Dye

BSA (Sigma) was conjugated to IRDye 800CW using the Protein Labeling Kit-High MW (catalog # 928–38040, LI-COR Biosciences, Lincoln, NE) according to the manufacturer’s instructions. The N-hydroxysuccinimide (NHS) ester reactive group of the 800CW dye couples free amino groups to form stable conjugates with proteins. Briefly, BSA (10 mg) was dissolved in 10 ml phosphate buffered saline (PBS). After adjusting the pH to 8.5, 1.0 mg of BSA was mixed with an appropriate amount of dye at 20°C for 2 h, protecting the reaction from light. Zeba™ desalting spin columns (Pierce, Rockford, IL) were used to separate free dye from the protein conjugate. The concentration of BSA-conjugated 800CW dye and the dye to protein ratio were determined by measuring absorbance with a spectrophotometer.

### *In vivo* and *ex vivo* optical imaging

A Pearl® Impulse Small Animal Imaging System (LI-COR) was used for i*n vivo* imaging. The instrument is designed specifically for white light, 700-nm channel, and 800-nm channel image acquisition. After the initial control (D0) scan, 1 nmol of each dye was administered to each mouse by tail vein injection; each mouse received only a single injection. Dynamic images were acquired before, and also at 1 h, 4 h, 6 h, 12 h, 24 h, then at every 24 h (till Day 14) after administration of the fluorescent probe (N = 4–5 for each group). All images were acquired with the same parameter settings and are scaled to the same maximum values. Estimates of probe biodistribution were determined based on the indicated circular regions of interest encompassing each kidney, and the average (arbitrary units)/area signal intensity was calculated using the manufacturer’s software. At each time point, the mice were anesthetized using isoflurane/oxygen through a nose cone attached to the imaging bed.

#### *ex vivo* organ imaging

*Ex vivo* organ imaging was conducted at 36 h after administration of the contrast agents using the Maestro^TM^ imaging system (CRi, Waltham, MA). Nephritis-induced mice from the RGD probe group and the control probe group were sacrificed immediately after imaging at 36 h (N = 3 for each group). Several organs were collected and washed with PBS, then laid on Petri dishes for imaging. Image processing and data analysis were performed using the DyCE software provided with Maestro^TM^ 2.10.0. Briefly, a spectrum of the background signal (peak emission, 770 nm) was first obtained from a mouse before injecting the RGD 800CW dye, while the spectrum of the RGD 800CW dye (peak emission, 800 nm) was detected using a solution of the dye in PBS (0.1 nmol/ml). The spectra were then imported and used to unmix the NIR dye signal from the background signal for *ex vivo* studies. The average signal was presented as (arbitrary units)/area.

### Statistical Analysis

Data are expressed as mean ± SEM. A two-tailed *t* test was performed to compare the difference between the two groups using SPSS 8.0 for Windows (SPSS Inc., Chicago, IL). A *P* value less than 0.05 was considered as statistically significant.

## Results

### Renal dysfunction in anti-GBM nephritis

**Figure 1:**
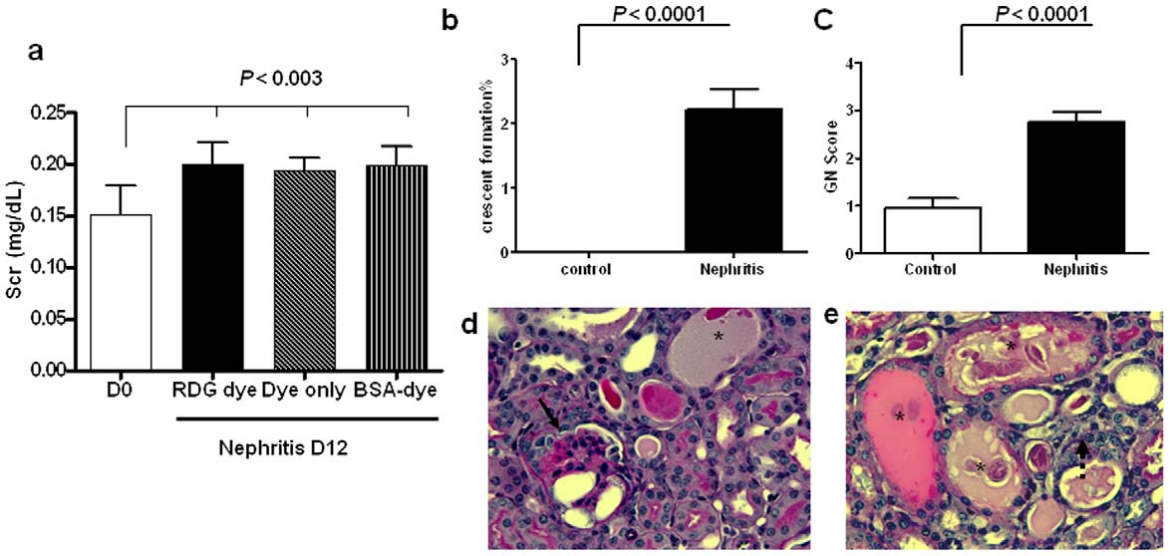
Renal dysfunction and pathological damage following anti-GBM nephritis. (*a*) Increased Scr after anti-GBM serum challenge. On D12, each nephritis group showed significantly higher levels of Scr than that on D0 (N = 4–5 for each group, *P*<0.003), whereas, there was no significant difference in Src levels among the three groups (*b–c*). Severe pathological damage was seen in the nephritis group on D12, as evidenced by crescent formation in the nephritis group and by an increased GN score (N = 3 for each group, *P*<0.0001). (*d–e*) Representative pathological changes in diseased mice in glomeruli (d) and tubulointerstitium are shown (e). The solid arrow indicates crescent formation; the asterisk indicates dilated distal tubules filled with protein cast; the dotted arrow shows infiltrating cells.

The 129x1/svJ mice challenged with anti-GBM serum showed increased levels of Scr post-challenge, accompanied by several renal pathological changes, including an increased glomerulonephritis (GN) score and crescent formation when compared to the no-disease control group (Fig. 1a–c). The three nephritis groups showed similar Scr levels on D12, indicating that all the study groups had similar renal function when recruited into the imaging study (Fig. 1a). Representative renal pathological changes in the glomeruli and the tubulointerstitium are shown in Figs. 1d and 1e, respectively.

### Increased expression of integrin αvβ3 in anti-GBM nephritis kidney

**Figure 2:**
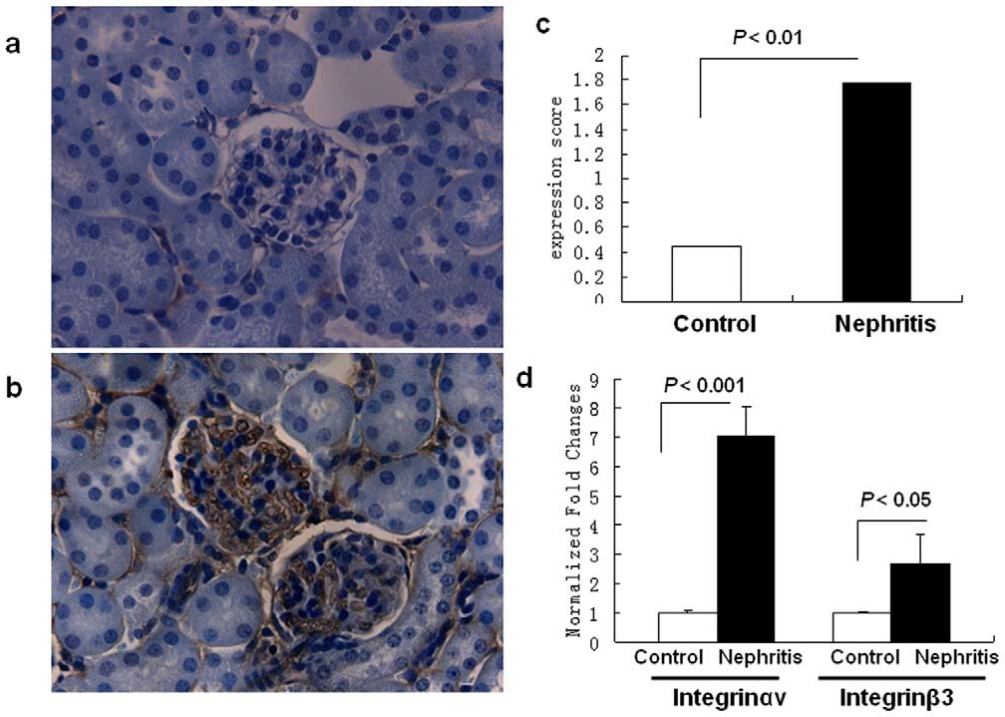
Increased renal integrin αvβ3 expression in anti-GBM disease. (*a–c*) IHC staining of mouse kidney sections with an anti-integrin αvβ3 antibody (1:500 dilution). (*a*) Normal control kidney; (*b*) nephritic kidney subjected to anti-GBM disease; (*c*) Semi-quantitative analysis of integrin αvβ3 expression (N = 6 for each group, *P*<0.01, two-tailed *t* test). (*d*) Real-time PCR analysis showing enhanced integrin αv and β3 gene expression in diseased kidney after anti-GBM serum injection (N = 6 for each group, *P*<0.001 for αv and *P*<0.05 for β3 gene expression, respectively, two- tailed *t* test).

In agreement with previous studies, IHC and qPCR findings confirmed the increased expression of αvβ3 in the diseased kidney at both the protein and mRNA levels following anti-GBM antibody challenge [19]. As shown in Fig. 2a, higher expression of αvβ3 was seen in the anti-GBM kidney tissue, predominantly in the mesangial areas and capillary loops. The mRNA expression of integrin αvβ3, normalized to that of cyclophilin A-2, is shown in Fig. 2d. The mRNA level of integrin αv in the nephritic tissue was 7 times higher than that in the control tissue (N = 6, *P*<0.001), whereas the mRNA level of integrin β3 gene was 2.7 fold higher than in controls (N = 6, *P*<0.05).

### *In vivo* renal dynamic imaging captured by the Pearl® Impulse system

**Figure 3:**
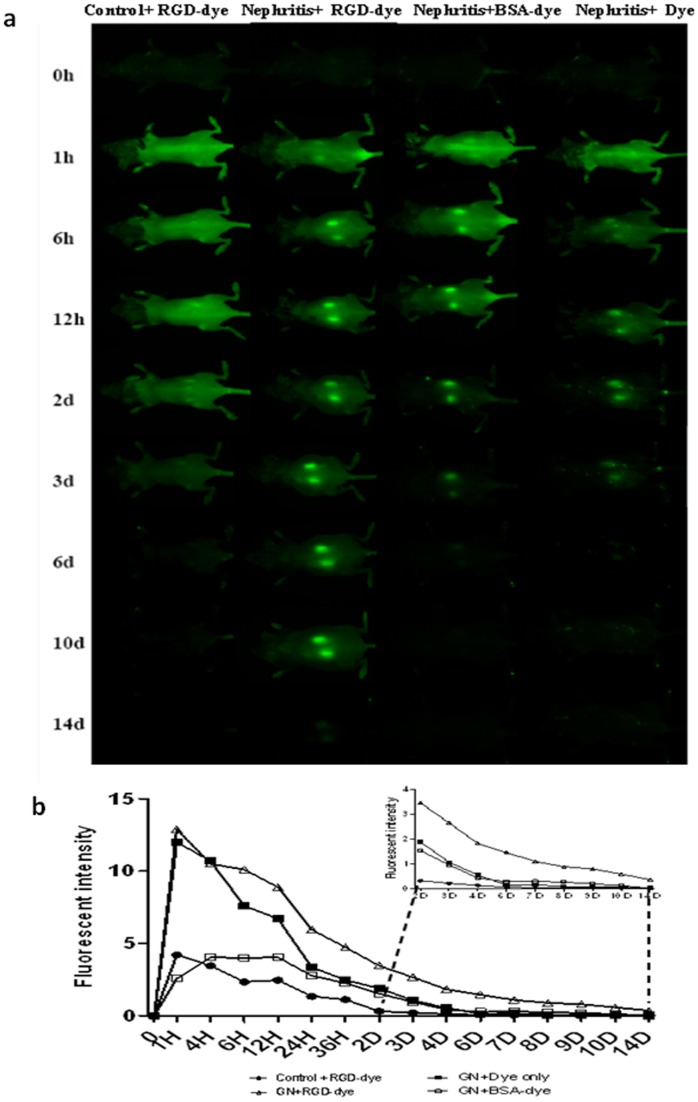
Dynamic *in vivo* fluorescence imaging using the Pearl® Impulse system. Repeated *in vivo* fluorescence imaging was performed using the Pearl® Impulse system over a period of 14 days after 800CW-RGD dye injection. (a) Dynamic renal imaging at successive time points. From left to right: healthy-control + RGD Dye, nephritis + RGD Dye, nephritis + BSA-conjugated dye, and nephritis + Dye only. (b) Average fluorescent signal intensity at each time point for each group, with the data from days D2–D14 shown in the inset (N = 4–5 for each group at each time point, *P<*0.05 for nephritis + RGD Dye group *vs*. other group, two-tailed *t* test). Note: “nephritis” refers to mice that have been challenged with anti-GBM serum.

Following administration of the RGD probe, normal control mice exhibited a weak, transient accumulation of the dye in the kidney around 6–24 h (Fig. 3a, left-most column), which was washed out by Day 2. In nephritis-induced mice injected with the unconjugated dye alone, some signal was observed in the kidney area up to 48 h compared to that in the normal control mice (average signal 1,890±0.124 *vs*. 0.312±0.054, N = 4 – 5 for each group, *P<*0.001, Fig. 3a, right most column). In nephritis-induced mice that received the RGD probe (coupled to dye), strong renal images can be acquired as early as 1 h after injection, and retained up 10 days after administration. The control group injected with BSA-conjugated to the dye behaved very similarly to the nephritic mice receiving free dye; renal images could be acquired at 6 h after administration of the dye and lasted for about 2 days (Fig. 3a, third column). The quantitative data for each group is shown in Fig. 3b, which clearly demonstrates the significantly higher fluorescence signal observed in the nephritis + RGD probe group, even towards the end of the experiment (D9–D14 after dye administration), compared to the other three groups.

### *Ex vivo* organ imaging using the Maestro^TM^ imaging system

**Figure 4:**
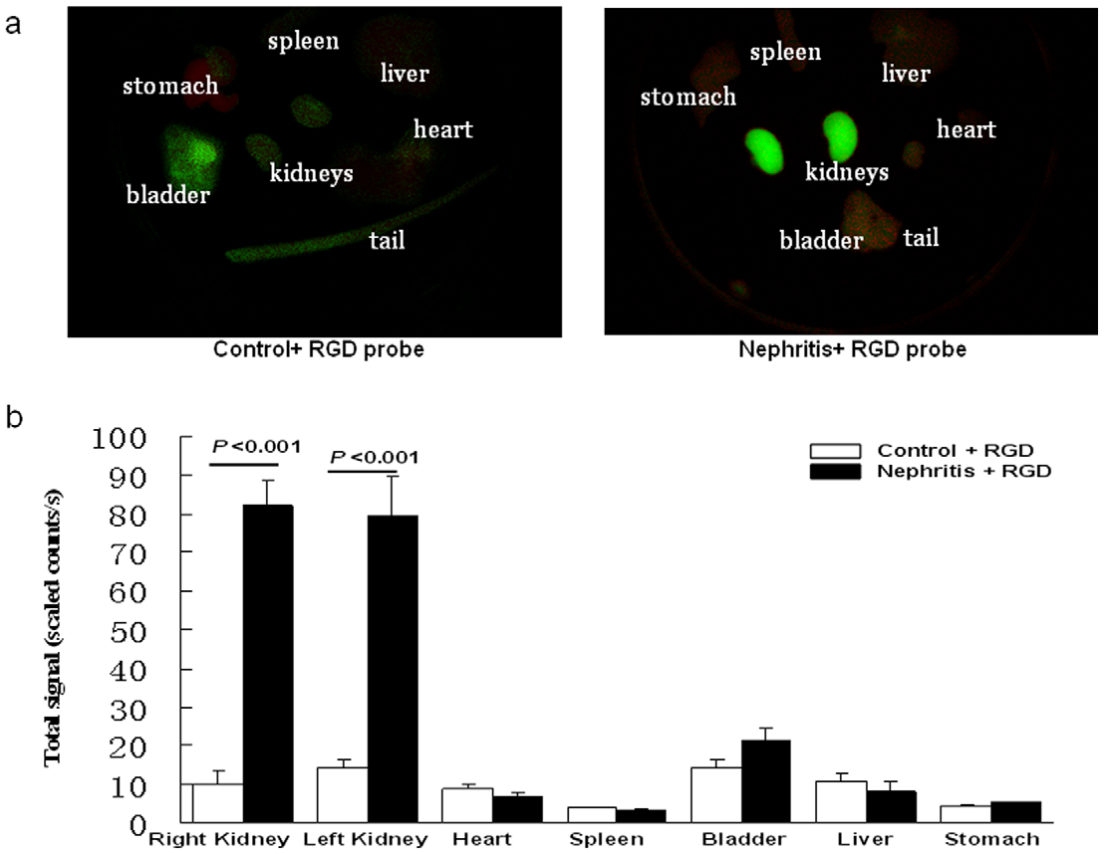
*Ex vivo* fluorescence imaging of organs using the Maestro^TM^ imaging system. (a) Three mice from either the nephritis (i.e., with anti-GBM disease) or the healthy-control group were sacrificed 36 h after administration of RGD dye. Kidneys, heart, liver, bladder, stomach, and tail tissue were placed in Petri dishes. Representative images captured by the Maestro^TM^ imaging system are shown. (b) The average fluorescent signal intensity of each organ is shown (N = 3 for each group, *p<*0.01 in kidney of the nephritis + RGD dye group *vs*. kidney of the healthy-control + RGD dye group).

Examination of isolated organs (kidney, heart, spleen, liver, stomach, bladder, and tail) from nephritic and control mice 36 h after RGD probe administration (N = 3 for each group) showed much higher signal in the kidneys from the nephritic group (Fig. 4a, b), using a second optical imaging platform, the Maestro^TM^ imaging system.

### Correlation of renal function changes with renal RGD accumulation

**Figure 5:**
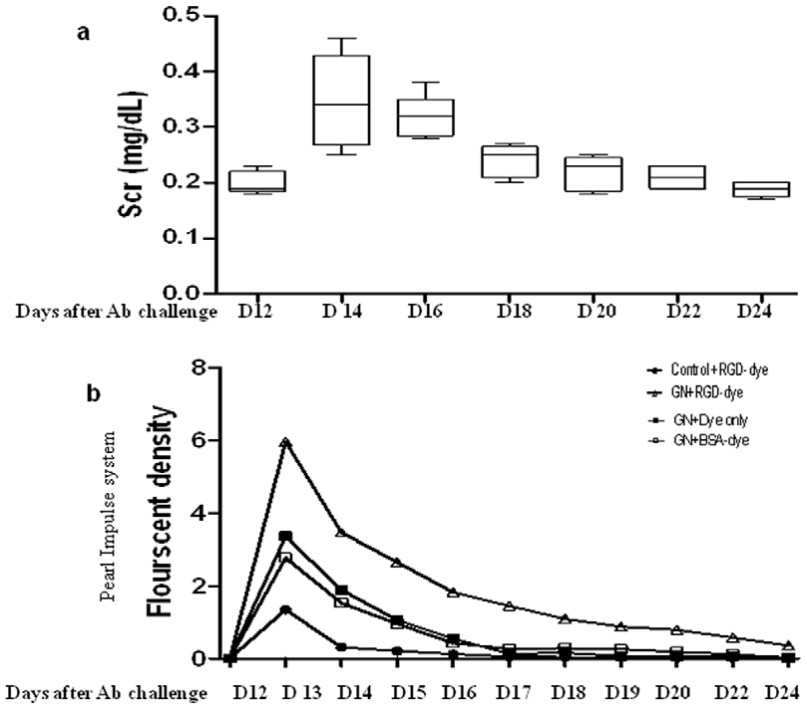
Correlation of renal function changes in anti-GBM disease with renal RGD probe accumulation in serial images. (a) The Scr levels were assayed every 2 days, and the longitudinal changes in Scr levels are plotted. A peak corresponding to high Scr levels was observed around D13–D19 after anti-GBM disease induction. The longitudinal accumulation of the renal RGD probe, as captured by the Pearl® Impulse system (b) is plotted in phase with the serum creatinine changes.

As shown in Fig. 1, imaging was initiated on D12 following anti-GBM challenge, when Scr levels are elevated in this model. Following this, changes in Scr levels were monitored every other day till the end of imaging. As indicated by the longitudinal changes in Scr levels in Fig. 5a, the disease peaked around D13 to D19 after anti-GBM serum injection. Following this, the mice partly recovered, with a gradual decrease in Scr levels. The renal fluorescent intensity changes, as captured by the Pearl® Impulse small-animal imaging system show that the renal accumulation of the RGD 800CW dye peaked just before D14 of disease, and then gradually dampened beyond that (Fig. 5b), closely tracking renal function as gauged by Scr levels (Fig. 5a).

## Discussion

We have performed *in vivo* and *ex vivo* renal imaging using a RGD-labeled NIR dye (800CW) using two different optical imaging systems. Our data indicate that the strong interaction between integrin αvβ3 and its peptide ligand, RGD, can be used to non-invasively monitor renal disease progression in an anti-GBM nephritis model. In addition, our study implicates a potential role for imaging-guided disease monitoring for renal diseases with high integrin αvβ3 expression.

Integrin αvβ3, also known as the vitronectin receptor, consists of a 125 kD αv subunit and a 105 kD β3 subunit. It is expressed at low levels in most normal tissues, but, of particular interest, highly expressed in inflamed sites and on activated macrophages. In various forms of glomerulonephritis, including IgA nephropathy, lupus nephritis, membranoproliferative glomerulonephritis, as well as diabetic nephropathy, high Integrin αvβ3 expression has been reported [17]–[21]. In patients with anti-GBM nephritis, an abnormal pattern of integrin αvβ3 expression was also consistently found on crescentic cells [19], [29]. Besides its role in the trafficking of inflammatory cells, this integrin may also function through other mechanisms. Several studies focus on the interaction of integrin αvβ3 with a variety of extracellular matrix components. Integrin αvβ3 has been identified as a key player in tubulo-interstitial nephritis, where it impacts tubule cell survival and proper tubular homeostasis [30]. The adhesive crosstalk between glomerular mesangial cells and fibrinogen is also integrin αvβ3 dependent [31]. Recently, Hayashida T *et al* also reported that Integrin αvβ3 can promote renal fibrogenesis through Rac-1 mediated ERK activity [32]. Thus, integrin αvβ3 may contribute to the tissue damage seen in various renal diseases through its crosstalk with a variety of extracellular matrix components. In rheumatoid arthritis, a disease characterized by inflammatory arthritis, blocking the function of integrin αvβ3 can significantly reduce inflammatory cell infiltration and restore joint function [33].

The recognition motif, RGD (Arg-Gly-Asp), is a tripeptide sequence that binds integrin αvβ3 receptors. Interest in using a labeled RGD peptide ligand for the study and/or monitoring of diseases related to the αvβ3 receptor over-expression is increasing. Several groups have labeled RGD successfully with fluorescent dyes for *in vitro* and *in vivo* imaging, mostly in tumor-related diseases [22], [23], [34]. These studies unanimously support the notion that the interaction between integrin αvβ3 and RGD can be used to study and/or monitor the progression of diseases with enhanced integrin αvβ3 expression. In agreement with previous studies, our IHC and qPCR findings demonstrated the overexpression of integrin αvβ3 in the renal tissue following anti-GBM nephritis induction, rendering it a facile tool for imaging.

To the best of our knowledge, no *in vivo* optical renal imaging based on integrin αvβ3 has been reported to date. One possible reason is that in the visible electromagnetic spectrum, tissue absorption and light scattering result in small penetration depths of a few millimeters. This makes the use of fluorescent agents in the visible spectrum suboptimal for deep organ imaging, including the imaging of the kidney. However, NIR fluorophores (700–900 nm), which have high molar extinction coefficients, good quantum yields, and low non-specific tissue binding can penetrate organs to a depth of several centimeters. Hence, these agents make deep organ *in vivo* imaging possible [11]–[14], [35]. Recently, Nakamura *et al*. demonstrated that Cy7-labeled recombinant-gelatin can be used to monitor renal inflammation [16]. However, this study was not performed in a true “*in vivo*” setting because the images were captured after the skin was removed.

The RGD 800CW probe created by LI-COR Biosciences is a NIR fluorescent agent that can bind specifically to integrin, with predominant renal excretion. We were able to successfully set up *in vivo* optical imaging based on this probe using the Pearl® Impulse small-animal imaging system. Our study shows that there is significantly higher accumulation of the RGD probe within nephritic kidneys, and that the accumulation can persist as long as 14 days following injection, whereas there is only a transient, low dye signal in the nephritic mice receiving the 800CW dye alone or the BSA-conjugated 800CW dye. These findings were consistent in both the *in vivo* and *ex vivo* imaging studies using two independent imaging platforms. In the healthy control group with no disease, we believe that the RGD probe is rapidly washed out after administration because there is no evidence of a renal-enhanced fluorescent signal captured at any time point.

Importantly, the change in the disease course was paralleled by the change in dye accumulation in the diseased kidney. As shown in Fig. 5, the high disease peak in our mouse model is around D13–D19 after anti-GBM serum challenge, as evidenced by increased Scr levels. The longitudinal changes in the fluorescent signal captured by the Pearl® Impulse imaging system (Fig. 5b) suggest that the high peak of the fluorescent signal in the nephritis group is around D12–D19 (D1–D7 after injection of the RGD probe), although the signal decreases slowly following this.

In contrast to CT and MRI, molecular and cellular information offered by molecular imaging approaches such as optical imaging may permit the earlier diagnosis of diseases, and quantitative monitoring of disease progression [12], [13], [35]. Optical fluorescent imaging has unique advantages including high picomolar molecular sensitivity, absence of ionizing radiation, relatively low cost and the possibility of using it in many modalities with different scales, when compared to other molecular imaging technologies [11]–[13], [35], [36]. The clinical translation of optical NIR imaging has been successfully reported in oncology practice, in which intra-operative fluorescence imaging can be used to detect sentinel lymph node involvement in patients with cervical and vulval cancer [37] or to guide surgical resection [36], as well as in differentiating between malignant and benign masses in the breast [38]. Moreover, NIR fluorescent dyes can be conjugated with antibody, peptide, or other small molecule, which allows for specific binding to specific target organs or tissues for potential therapeutic or diagnostic monitoring. Hence, we believe that NIR imaging is likely to emerge as a powerful tool for monitoring renal disease in the near future.
